# Epidemiology of musculoskeletal upper extremity ambulatory surgery in the United States

**DOI:** 10.1186/1471-2474-15-4

**Published:** 2014-01-08

**Authors:** Nitin B Jain, Laurence D Higgins, Elena Losina, Jamie Collins, Philip E Blazar, Jeffrey N Katz

**Affiliations:** 1 Department of Physical Medicine and Rehabilitation, Spaulding Rehabilitation Hospital and Harvard Medical School, Boston, MA, USA; 2 Department of Orthopaedic Surgery, Brigham and Women’s Hospital and Harvard Medical School, Boston, MA, USA; 3 Harvard Shoulder Service, Harvard Medical School, Boston, MA, USA; 4 Division of Rheumatology, Immunology, and Allergy, Brigham and Women’s Hospital and Harvard Medical School, Boston, MA, USA; 5 Department of Biostatistics, Boston University School of Public Health, Boston, MA, USA; 6 Orthopedic and Arthritis Center for Outcomes Research, Brigham and Women’s Hospital, 75 Francis Street, BC-4-016, Boston, MA 02115, USA

**Keywords:** Rotator cuff repair, Arthroscopy, Utilization, Epidemiology

## Abstract

**Background:**

Musculoskeletal disorders of the upper extremity are common reasons for patients to seek care and undergo ambulatory surgery. The objective of our study was to assess the overall and age-adjusted utilization rates of rotator cuff repair, shoulder arthroscopy performed for indications other than rotator cuff repair, carpal tunnel release, and wrist arthroscopy performed for indications other than carpal tunnel release in the United States. We also compared demographics, indications, and operating room time for these procedures.

**Methods:**

We used the 2006 National Survey of Ambulatory Surgery to estimate the number of procedures of interest performed in the United States in 2006. We combined these data with population size estimates from the 2006 U.S. Census Bureau to calculate rates per 10,000 persons.

**Results:**

An estimated 272,148 (95% confidence intervals (CI) = 218,994, 325,302) rotator cuff repairs, 257,541 (95% CI = 185,268, 329,814) shoulder arthroscopies excluding those for cuff repairs, 576,924 (95% CI = 459,239, 694,609) carpal tunnel releases, and 25,250 (95% CI = 17,304, 33,196) wrist arthroscopies excluding those for carpal tunnel release were performed. Overall, carpal tunnel release had the highest utilization rate (37.3 per 10,000 persons in persons of age 45–64 years; 38.7 per 10,000 persons in 65–74 year olds, and; 44.2 per 10,000 persons in the age-group 75 years and older). Among those undergoing rotator cuff repairs, those in the age-group 65–74 had the highest utilization (28.3 per 10,000 persons). The most common indications for non-cuff repair related shoulder arthroscopy were impingement syndrome, periarthritis, bursitis, and instability/SLAP tears. Non-carpal tunnel release related wrist arthroscopy was most commonly performed for ligament sprains and diagnostic arthroscopies for pain and articular cartilage disorders.

**Conclusions:**

Our data shows substantial age and demographic differences in the utilization of these commonly performed upper extremity ambulatory procedures. While over one million upper extremity procedures of interest were performed, evidence-based clinical indications for these procedures remain poorly defined.

## Background

Musculoskeletal disorders of the upper extremity are common reasons for patients to seek care and undergo ambulatory surgery [1-3]. Ambulatory surgery was introduced because it generally costs less than inpatient surgery and because technological advancements have enabled a safe transition from the in-patient setting [4,5]. The number of procedures performed in ambulatory surgery centers increased from 380,000 in 1983 to 31.5 million in 1996 and to 57.1 million in 2006 [4,6,7]. It was recently reported that 272,148 rotator cuff repair and 577,000 carpal tunnel release procedures were performed in the United States in 2006 on an ambulatory basis [1,2]. The annual national utilization of shoulder arthroscopy performed for indications other than rotator cuff tear and wrist arthroscopy performed for indications other than carpal tunnel syndrome have not been previously reported.

The National Survey of Ambulatory Surgery (NSAS) is a Center for Disease Control and Prevention (CDC) based population sample survey of ambulatory surgery procedures performed in the United States [8]. The NSAS databases were assembled in 1994–96 and not again until 2006. Thus the 2006 NSAS databases permit contemporary estimates of upper extremity orthopedic surgery. We sought to compare utilization, demographics, indications, and operating room time for most commonly performed ambulatory procedures of the upper extremity. This data is helpful in comparing commonly performed upper extremity orthopedic procedures and may also assist policy makers and hospitals in projecting future needs to accommodate upper extremity procedural volume.

## Methods

### Database description

The NSAS [8] includes ambulatory surgery procedures performed in hospitals and freestanding ambulatory surgery centers. The NSAS is available for download via a file transfer protocol (FTP) server [9]. A multi-stage probability design was used to sample facilities [10]. Facilities were stratified by facility type (hospital versus freestanding), ambulatory surgery status of hospitals, facility specialty, and geographic region [10]. Within sampled facilities, a sample of ambulatory surgery visits were selected using a systematic random sampling procedure [10]. Data were then abstracted from the medical record for each sampled visit [10]. In comparison to the 2006 NSAS, the 1994–1996 NSAS used a stratified cluster sampling design.

The NSAS was validated by an independent contractor [6]. As per information from NSAS [6], approximately 10 percent of the abstractions were independently recoded. The overall error rate did not exceed 0.3% for coding and keying of diagnosis, procedure, and demographic information.

The NSAS has a total of 52,233 records that correspond to approximately 34.7 million ambulatory surgery visits in the United States in the year 2006. The 2006 sample included 189 eligible hospitals and 397 eligible ambulatory surgery centers. Of these, 142 (75%) hospitals and 295 (74%) ambulatory surgery centers responded.

### Sample selection

Each record in the NSAS contains seven possible diagnosis and six possible procedure codes based on the International Classification of Diseases, 9^th^ Revision, Clinical Modification (ICD-9-CM). We selected records with any ICD-9-CM procedure code for rotator cuff repair (83.63), shoulder arthroscopy (80.21), elbow arthroscopy (80.22), carpal tunnel release (04.43), and wrist arthroscopy (80.23). Patients with any procedure code for rotator cuff repair were excluded from our estimates of shoulder arthroscopy not performed for cuff repairs. Similarly, patients with any procedure code for carpal tunnel release were excluded from our estimates of wrist arthroscopy not performed for carpal tunnel release.

Our final sample included records of rotator cuff repair (n = 407), shoulder arthroscopy performed for indications other than cuff repair (henceforth referred to as shoulder arthroscopy; n = 466), elbow arthroscopy (n = 10), carpal tunnel release (n = 1,028), and wrist arthroscopy performed for indications other than carpal tunnel repair (henceforth referred to as wrist arthroscopy; n = 69).

### Demographic and clinical variables examined

We analyzed age, sex, principal source of payment (classified as Medicare, Medicaid, private or commercial insurance, Workers Compensation, and other such as TRICARE [11], government, self-pay, charity care or write-off). The age distribution was based on an *a priori* understanding of the pathophysiology of shoulder and wrist disorders that may require ambulatory surgery. Rotator cuff disorders are usually secondary to trauma in patients younger than 45 years of age and are degenerative in patients older than 45 years [12]. Wrist disorders such as carpal tunnel have been described in post-menopausal women [13,14] and in patients with repetitive wrist motions that occur in patients in the working age-groups [15,16]. It is also of importance to understand upper extremity procedures performed in patients 75 years and older given the increasing proportion of persons in this age-group in the United States.

In comparison to the 1994–1996 NSAS that did not provide information on nerve block location, the 2006 NSAS provides information on the type of anesthesia used during a surgical procedure including whether the patient received a nerve block. Operating room time was the total time spent in the operating room by the patient. Length of surgery extended from when the first incision was made until the wound was closed. Indications for shoulder and wrist arthroscopy were ascertained based on primary ICD-9-CM diagnoses codes. These indications were categorized as: shoulder instability (718.81), Superior Labrum Anterior and Posterior (SLAP) lesions (840.7), other shoulder disorders with an ICD-9-CM diagnosis code of 726.2, sprains and strains of the wrist (842.0), wrist pain (719.43), and articular cartilage disorders of the wrist (718.03). ICD-9-CM codes are stratified by body-part and include wrist as part of the forearm. Hence, codes that were specific to the forearm were assumed to be for the wrist in our study since patients in our study underwent wrist arthroscopy.

### Statistical analysis

We estimated the population-based number of upper extremity procedures of interest performed in the United States in 2006 by using sampling weights provided in NSAS. The sampling weights in NSAS have three components (inflation by reciprocals of the probabilities of sample selection, adjustment for non-response, and population weighting ratio adjustments) that produce essentially unbiased national estimates [6]. We calculated 95% confidence intervals around the point estimates using strata and cluster variables that are commonly used survey sampling techniques [17,18]. We calculated age-stratified procedure utilization rates by using the U.S. Census Bureau 2000-based postcensal estimates of the civilian population as of July, 1, 2006 for age-groups 15 years and older [19]. The age-stratified rates were calculated per 10,000 persons. We also present unweighted proportion of procedures performed stratified by sex, primary payer, facility type (hospital-based versus freestanding), and select clinical indications.

Population estimates calculated based on fewer than 60 records in NSAS and estimates that have a standard error exceeding 30 percent of the point estimate are considered unreliable and noted in the tables as such [10].

We performed statistical analyses using SAS for Windows (version 9.2), SAS Institute Inc., (Cary, NC). Ethics approval for this study was obtained from the Partners Human Research Committee.

## Results

### Estimated upper extremity ambulatory procedures

An estimated 272,148 (95% CI = 218,994, 325,302) patients underwent rotator cuff repair, 257,541 (95% CI = 185,268, 329,814) patients underwent shoulder arthroscopy, 3,686 (95% CI = 3,554, 3,818; estimates unreliable) patients underwent elbow arthroscopy, 576,924 (95% CI = 459,239, 694,609) patients underwent carpal tunnel release, and 25,250 (95% CI = 17,304, 33,196) patients underwent wrist arthroscopy in the United States in the year 2006 (Table 1). Due to the small number of elbow arthroscopy procedures, these procedures were not analyzed further.

**Table 1:** Estimates of upper extremity ambulatory surgery in the United States, 2006

**Procedure**	**Number of procedures**	**95% confidence intervals**
Rotator cuff repair	272,148	218,994, 325,302
Shoulder arthroscopy*	257,541	185,268, 329,814
Elbow arthroscopy	3,686	3,554, 3,818
Carpal tunnel release	576,924	459,239, 694,609
Wrist arthroscopy*	25,250	17,304, 33,196

^*^Shoulder arthroscopy excludes rotator cuff tears; Wrist arthroscopy excludes carpal tunnel syndrome.

### Demographic characteristics

Shoulder and wrist arthroscopy were more commonly performed in younger patients who were 15–44 years of age, with 39% of shoulder arthroscopies and 60% of wrist arthroscopies performed in this age group (Table 2). Fifty eight percent of rotator cuff repairs and 50% of carpal tunnel releases were performed in 45–64 year old adults. Patients in the age-group 65–74 years comprised 20% of all rotator cuff repairs and 12% of all carpal tunnel releases performed in 2006. Carpal tunnel release was predominantly performed in females (68%) whereas shoulder arthroscopy was more often performed in males (60%). When the data were stratified by age and sex, most patients in the younger age-group of 15–44 years undergoing cuff repairs and shoulder arthroscopy were male (72% for cuff repairs and 63% for shoulder arthroscopy).

**Table 2:** Demographic characteristics of patients undergoing ambulatory surgery for upper extremity in the United States, 2006

**Characteristics**	**Rotator cuff repair**	**Shoulder arthroscopy**^**╪**^	**Carpal tunnel release**	**Wrist arthroscopy**^**╪**^
**Age (years)**				
15-44	14%	39%	26%	60%
45-64	58%	50%	50%	32%
65-74	20%	6%	12%	6%
≥75	8%	4%	11%	-
**Sex**				
Female	44%	40%	68%	42%
Male	56%	60%	32%	58%
**Primary payer**				
Medicare	23%	9%	24%	2%
Medicaid	2%	1%	3%	-
Private or commercial insurance	55%	65%	58%	62%
Workers compensation	16%	20%	13%	37%
Other*	4%	5%	2%	-
**Facility type**				
Hospital-based	40%	27%	22%	18%
Freestanding	60%	73%	78%	82%
Anesthesia**				
General	85%	86%	20%	66%
Block	21%	22%	29%	32%
Topical/local	6%	6%	28%	16%
Intravenous sedation	10%	7%	33%	11%

^**╪**^Shoulder arthroscopy excludes rotator cuff tears; Wrist arthroscopy excludes carpal tunnel syndrome.

*Other includes TRICARE, other government, self pay, charity care/ write off.

**Total does not equal a 100% since types of anesthesia are not mutually exclusive and data for other types of anesthesia such as intravenous, epidural, etc. are not presented.

Number of subjects missing in non-weighted data for primary payer = 6 for rotator cuff repair, 4 for shoulder arthroscopy, 9 for carpal tunnel release, and 2 for wrist arthroscopy.

Table represents unweighted proportions.

The majority (55%-65%) of all cases were primarily paid for by private or commercial insurance. Workers Compensation was the primary payer in 37% of wrist arthroscopy cases and 20% of shoulder arthroscopy cases. Among patients paid for by Workers Compensation, males comprised the majority undergoing rotator cuff repair (63%), shoulder arthroscopy (65%), and wrist arthroscopy (68%). In contrast, most patients paid for by Workers Compensation undergoing carpal tunnel release were females (63%).

### Age-stratified utilization rates

Of the procedures assessed in this study, carpal tunnel release had the highest utilization rate (44.2 per 10,000 persons in the age-group 75 years and older; 38.7 per 10,000 persons in 65–74 year olds, and; 37.3 per 10,000 persons in persons of age 45–64 years; Table 3). Among persons undergoing rotator cuff repairs, those in the age-group 65–74 had the highest utilization (28.3 per 10,000 persons). This was followed by persons in the age-group of 45–64 years (21.1 per 10,000 persons) and those in the age-group ≥75 years (11.8 per 10,000 persons). Shoulder arthroscopy had the highest rate in the age-group 45–64 years (17.1 per 10,000 persons), followed by age-group 65–74 years (9.0 per 10,000 persons), and age-group 15–44 years (7.9 per 10,000 persons).

**Table 3:** Age and sex stratified estimated utilization rates of ambulatory surgery for upper extremity per 10,000 persons in the United States, 2006

**Rotator cuff repair**			
**Age-group**	**Rate per 10,000 persons**	**Rate per 10,000 males**	**Rate per 10,000 females**
	**(95% confidence intervals)**	**(95% confidence intervals)**	**(95% confidence intervals)**
15-44	3.1 (1.6 – 4.5)	4.7 (1.9 – 7.6)^┼^	1.3 (0.5 – 2.2)^┼^
45-64	21.1 (16.7 – 25.6)	23.3 (17.0 – 29.7)	19.0 (12.9 – 25.2)
65-74	28.3 (13.3 – 43.3)	31.3 (12.9 – 49.6)^┼^	25.8 (10.4 – 41.2)^┼^
≥75	11.8 (5.8 – 17.7)^┼^	12.3 (4.9 – 21.9)^┼^	11.4 (4.0 – 18.9)^┼^
**Shoulder arthroscopy (Other than for rotator cuff tear)**			
15-44	7.9 (5.0 – 10.9)	8.6 (5.6 – 11.7)	7.2 (3.7 – 10.6)
45-64	17.1 (12.0 – 22.2)	19.6 (12.5 – 26.7)	14.7 (8.2 – 21.3)
65-74	9.0 (1.8 – 16.1)^┼^	12.6 (0.0 – 27.6)^┼^	5.9 (2.2 – 9.6)^┼^
≥75	6.6 (2.4 – 10.7)^┼^	3.8 (0.0 – 7.7)^┼^	8.2 (1.8 – 14.7)^┼^
**Carpal tunnel release**			
15-44	11.3 (7.5 – 15.1)	8.1 (4.9 – 11.2)	14.7 (9.2 – 20.2)
45-64	37.3 (27.5 – 47.1)	21.5 (13.6 – 29.3)	52.4 (38.2 – 66.6)
65-74	38.7 (26.1 – 51.2)	30.3 (10.9 – 49.7)^┼^	45.7 (29.4 – 62.0)
≥75	44.2 (26.0 – 62.5)	32.4 (17.1 – 47.7)^┼^	51.5 (24.6 – 78.4)

Rates were calculated using weighted procedure estimates and the U.S. Census Bureau 2000-based postcensal estimates of the civilian population as of July, 1, 2006.

Sample size for wrist arthroscopy too small to calculate reliable rates.

^┼^Estimates may be unreliable.

### Clinical characteristics of upper extremity ambulatory surgery

**Figure 1:**
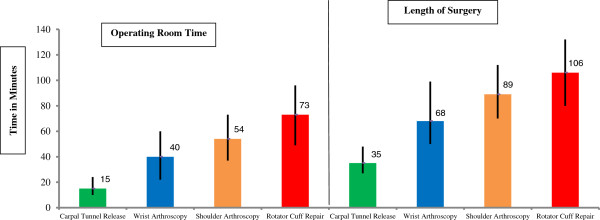
Length of surgery* and operating room time* for upper extremity ambulatory procedures in the United States, 2006.

Shoulder arthroscopy was performed for a primary diagnosis of impingement syndrome or periarthritis (34% of cases) and instability/Superior Labrum Anterior and Posterior (SLAP) lesions (13% of cases). Non-carpal tunnel release related wrist arthroscopy was most commonly performed for ligament sprains (36% of cases) and diagnostic arthroscopies for pain (12% of cases) and articular cartilage disorders (6% of cases). The median operating room time and surgical time utilized per procedure was greatest for rotator cuff repair (73 minutes for surgical time and 106 minutes for operating room time; Figure 1) and the least for carpal tunnel release (15 minutes for surgical time and 35 minutes for operating room time). The ratio of surgical time to operating room time was highest for rotator cuff repair (69%) and lowest for carpal tunnel release (43%). This ratio was 61% for shoulder arthroscopy and 59% for wrist arthroscopy.

## Discussion

We assessed the utilization of select upper extremity ambulatory procedures in the United States using a national database, NSAS, for the year 2006. We also studied age-stratified utilization rates, indications, and characteristics of patients undergoing these procedures. We found that over one million upper extremity ambulatory procedures of interest were performed. In general middle-aged adults in the age-group 45–64 years had the highest rate of these procedures, and with the exception of carpal tunnel release which had the highest rate in those 75 and older. Private or commercial insurance and Worker’s Compensation were the primary payers in a majority of cases. The most common indications for shoulder arthroscopy were impingement syndrome, periarthritis, and instability/SLAP tears whereas those for wrist arthroscopy were ligament sprains and cartilage disorders.

By way of comparison, in 2006, meniscal procedures of the knee (excision of semilunar cartilage) were performed on an estimated 690,000 cases [6] on an ambulatory basis. In the same year, 984,607 knee arthroscopies were performed [20]. Hence, the resources utilized by upper extremity procedures in the ambulatory setting are substantial and comparable to the most frequently performed orthopedic procedures in the ambulatory setting.

Our data only capture upper extremity procedures performed on an ambulatory basis. Data from the National Hospital Discharge Survey presented by the American Academy of Orthopaedic Surgeons estimated that in 2006 approximately 20,000 rotator cuff repairs were performed on an in-patient basis [21]. Thus, our estimate of 272,148 rotator cuff repairs per year may understate the true frequency by 7%. Similarly, an estimated 9,231 carpal tunnel release procedures were performed in 2006 as per data from the Nationwide Inpatient Samples [2]. Thus, our study likely underestimates the true frequency of carpal tunnel release by 1.6%. We are not aware of data on shoulder arthroscopy performed on ambulatory or non-ambulatory basis. However, a recent study using data from the American Board of Orthopaedic Surgery (ABOS) reported that 87.7% of Bankart repairs in 2006–2008 were performed arthroscopically [22], most likely in an ambulatory setting. Thus, it is likely that the majority of shoulder arthroscopies were captured by the ambulatory database used in our study.

As expected, the use of these upper extremity procedures varied across demographic groups. Shoulder arthroscopy was performed in 15–44 year olds at a higher rate as compared with rotator cuff repairs and wrist arthroscopy. This is likely because instability and SLAP lesions are most frequently operated on in young patients and in those who participate in organized sports due to a high rate of recurrent dislocation [23,24]. In our study, carpal tunnel release was most commonly performed in post-menopausal women consistent with several studies documenting the incidence of carpal tunnel syndrome to be highest in post-menopausal women [13,14]. In an Italian population, Mondelli et al. reported an incidence rate of carpal tunnel syndrome of 506 per 100,000 person-years for women, with peak incidence in women between 50–59 years of age [25]. Several reasons such as hormone replacement therapy [26,27], endogenous hormonal factors [27], presence of menstrual disorders [27], and increased expression of estrogen receptors in the tenosynovium [28] have been postulated to explain these findings. Workers Compensation was a primary payer in 13%-37% of upper extremity ambulatory cases. These work-related injuries were likely due to a traumatic event or from repetitive motion [29].

Shoulder arthroscopy was most commonly performed for a diagnosis of instability, SLAP lesions, and other disorders with an ICD-9 diagnosis code of 726.2. The increasing utilization of shoulder arthroscopy for management of instability [22] has likely enabled treatment of such patients on an ambulatory basis. As per communication with certified coders specializing in shoulder surgery from our institution [30], other disorders that are usually coded using the ICD-9 code 726.2 include impingement syndrome, periarthritis of the shoulder region, and scapulothoracic bursitis (likely subacromial/subdeltoid bursitis leading to subacromial decompression). Patients undergoing shoulder arthroscopy for reasons other than cuff repairs were younger, on average, than patients undergoing arthroscopic cuff repairs. This is expected since arthroscopic treatment for instability and SLAP lesions is usually performed in a younger population. Weber et al. reported that the mean age of male patients undergoing SLAP repairs was 37 years and that of female patients was 40.9 years based on data self-reported by candidates admitted to Part II of the American Board of Orthopedic Surgery Examination [31]. Despite the lack of evidence on treatment of SLAP lesions, Zhang et al. reported a steady increase in SLAP repairs from 2004–2009 in the United States based on billing records from several insurance companies [32]. Wrist arthroscopy was performed in a relatively smaller number of patients and most common indications included sprains and strains, wrist pain, and articular cartilage disorders. These diagnoses likely include sprain/tear of triangular fibrocartilage complex (TFCC), intercarpal ligament injuries, and synovitis that are common indications for wrist arthroscopy [33,34]. We cannot assess the prevalence of these specific indications since a specific ICD-9-CM diagnosis code is not assigned to these disorders. The high utilization of diagnostic wrist arthroscopies for pain and articular cartilage disorders is likely because MRI/MRA imaging correlation for wrist disorders is still not optimal [35]. TFCC injuries are one of the most common causes of wrist pain. In a specialty practice, Park et al. reported that 43% of patients with ulnar-sided wrist pain required arthroscopic intervention and all of these patients had a TFCC injury on intra-operative assessment [34]. Thus, a large proportion of patients with TFCC injuries require arthroscopic intervention and this likely comprises a substantial proportion of patients undergoing wrist arthroscopy in our study. Our data shows that as total operating room time increases, the ratio of actual surgical time (time from first incision until wound closure) to operating room time also increases. This could be interpreted as an increase in efficiency in the use of total operating time, with rotator cuff repair being the most efficient procedure by this criterion.

It is estimated that 40% of persons over the age of 50 years have cuff tears on imaging as compared with 54% in those >60 years, and 65% in persons over 70 years [36,37]. Thus, the prevalence of rotator cuff tears on imaging and cadaveric studies rises with increasing age. The prevalence of symptomatic rotator cuff tears across age is unknown. Our results show that the rate of rotator cuff repairs was highest in 45–74 year olds and declined in patients 75 years and older. It is possible that concerns about post-surgical cuff healing and rehabilitation potential of older patients discourages surgeons from recommending repair in these older persons [38,39]. It is also possible that tears are more symptomatic in younger persons who are traditionally more active. Moreover, patients in the older age-groups are likely not working, may perform less strenuous activities and may be less likely to opt for surgery. Further analysis of indications for surgery across age strata requires investigation.

The limitations of our study include the lack of data from Federal, military, and Department of Veteran’s Affairs hospitals. Therefore, our estimates are likely conservative. It is possible that some procedures were revision surgeries. This information cannot be ascertained from the database. Strengths of our data include a relatively large sample size and the ability to calculate national estimates but our data source lacks more detailed clinical information such as disease severity and patient outcomes.

## Conclusions

Rotator cuff repair, shoulder arthroscopy, carpal tunnel release, and wrist arthroscopy were performed an estimated 272,148 times, 257,541 times, 576,924 times, and 3,686 times, respectively, in the United States in the year 2006. The outcomes of rotator cuff repair and of non-operative management of these lesions in older patients needs further evaluation so that informed decisions about surgical intervention are made. The clinical and demographic predictors of the outcomes of many of these upper extremity ambulatory surgeries also remain ill-defined despite the substantial healthcare resource utilization associated with these procedures.

## Competing interests

Dr. Jain is supported by funding from National Institute of Arthritis and Musculoskeletal and Skin Diseases (NIAMS) project number 1K23AR059199, Foundation for PM&R, and Biomedical Research Institute at Brigham and Women’s Hospital. Drs. Katz and Losina are supported by NIAMS P60 AR 47782, T32 AR 055885 and Dr. Losina is supported by NIAMS K24 AR 057827. Ms. Collins is supported by NIAMS T32 AR 055885.

## Authors’ contributions

NJ participated in conception and design, acquisition of data, analysis, interpretation, and drafting the manuscript. LH participated in conception and design, acquisition of data, interpretation, and critical revision of the manuscript. JC participated in acquisition of data, interpretation, and critical revision of the manuscript. EL participated in conception and design, interpretation, and drafting the manuscript. PB participated in conception and design, interpretation, and critical revision of the manuscript. JK participated in conception and design, interpretation, and drafting the manuscript. All authors read and approved the final manuscript.

## Pre-publication history

The pre-publication history for this paper can be accessed here:

http://www.biomedcentral.com/1471-2474/15/4/prepub
